# Differences in microRNA levels across metabo-endotypes reveal novel insights into asthma heterogeneity

**DOI:** 10.1186/s12931-025-03452-x

**Published:** 2026-01-07

**Authors:** Rinku Sharma, Rachel S. Kelly, Kevin Mendez, Qingwen Chen, Julian Hecker, Sofina Begum, Clary Clish, Juan C. Celedón, Kelan G. Tantisira, Scott T. Weiss, Jessica Lasky-Su, Michael J. McGeachie

**Affiliations:** 1 Channing Division of Network Medicine, Brigham and Women’s Hospital and Harvard Medical School, 181 Longwood Ave., Boston, MA 02115 USA; 2 The Broad Institute of MIT and Harvard, Boston, USA; 3 Division of Pediatric Pulmonary Medicine, UPMC Children’s Hospital of Pittsburgh, Pittsburgh, USA; 4 Division of Pediatric Respiratory Medicine, University of California San Diego and Rady Children’s Hospital, San Diego, CA USA

**Keywords:** Asthma, MicroRNAs, GACRS, Metabolomics, Metabo-endotype

## Abstract

**Rationale:**

We previously validated five clinically distinct asthma metabo-endotypes (mechanistically derived asthma subgroups). We hypothesize that metabo-endotype membership may be partially driven by differences in serum microRNA profiles and their influence on metabolite levels.

**Objectives:**

To determine whether serum miRNA levels can help understand the underlying drivers of metabolic dysregulation across metabo-endotypes.

**Method:**

We compared expression levels of serum microRNAs across 1121 children grouped into five asthma metabo-endotypes using ANCOVA. A LASSO model was leveraged to determine the most important miRNAs for discriminating metabo-endotype membership. Finally, multiple linear regression models and two-sample t-tests were employed to determine whether serum microRNA ~ plasma metabolite relationships differed between individuals within different metabo-endotypes.

**Measurements and main results:**

Of 317 serum miRNAs, 132 (41.6%) demonstrated significantly different expression across metabo-endotypes (FDR < 0.05), with miR-143-3p showing the greatest variation (FDR *p* = 5.7 × 10^− 19^). Most differences were driven by metabo-endotypes 2 and 3, the most and least severe. Enrichment analysis of microRNAs’ predicted target genes revealed critical asthma pathways, including Th17 and Th1/Th2 cell differentiation. A model based on 17 miRNAs was able to discriminate membership of metabo-endotype 2 versus 3 (AUC:81%, CI: 73%-88%). There was some evidence that relationships between specific miRNAs and metabolites differed between individuals in metabo-endotypes 2 and 3, which may suggest differential posttranscriptional regulation of pathways including eicosanoid and arginine metabolism.

**Conclusions:**

The results provide some evidence to suggest differential miRNA regulated gene expression between biologically and clinically distinct asthma metabo-endotypes, with a potentially important role for miR-143-3p. Understanding these relationships may uncover novel therapeutic targets and guide more personalized treatment strategies.

**Supplementary Information:**

The online version contains supplementary material available at 10.1186/s12931-025-03452-x.

## Introduction

Asthma is a common chronic condition characterized by the heterogeneity in its clinical manifestations [1]. Efforts to disentangle this heterogeneity have recently moved towards classifying individuals with asthma into subgroups based on the underlying biological mechanisms of their condition, or ‘endotyping’ [2]. Endotyping is hypothesized to be more informative in terms of pathobiological understanding than symptom-based categorizations of asthma and may more directly aid the identification of novel targets for prevention and management. The metabolome, as the closest ‘ome to phenotype, reflecting both the upstream ‘omes, including genetics, and their interactions with the environment, is considered particularly informative in this regard [3]. As asthma is a whole-system disorder arising from the interactions between underlying genetic susceptibility and environmental exposures [4], and as asthma and its varying symptoms have been associated with metabolic dysregulation [5], metabo-endotypes are of increasing interest.

We previously identified and validated five “metabo-endotypes” of asthma that differed in metrics of clinical severity [6]. In a population of children with asthma from Costa Rica, we clustered the children into metabo-endotypes using their global plasma metabolomic profile. We defined one cluster of children, who demonstrated the poorest lung function, greatest use of oral steroids and inhaled β2-agonists, and highest eosinophil count as our “most-severe” metabo-endotype. The metabo-endotype with individuals who performed best in these categories was defined as our “least-severe”. We then recapitulated these metabo-endotypes in a population of North American children with asthma and were able to replicate these clinical differences. Further exploring the upstream drivers of the metabolite levels can help us to better understand the etiology of the resulting metabo-endotypes and to identify novel targets for their management.

MicroRNAs (miRNAs) are increasingly understood to function as key regulators of metabolism [3], and we have demonstrated direct associations between miRNAs and metabolites in the context of asthma phenotypes [7]. As such, we hypothesize that the metabolomic differences driving the clinical variation between our metabo-endotypes may be, in part, explained by differences in microRNA levels. In this study we leveraged serum microRNA data collected in our Costa Rican population and compared microRNA levels between individuals in our five metabo-endotypes. We further sought to identify relationships between microRNA levels and the key metabolites driving the formation of our endotypes.

## Methods

### Study population

The study population has previously been described. In brief, The Genetics of Asthma in Costa Rica Study (GACRS) [7–12] recruited 1,165 children with asthma aged 6–14 years from February 2001-July 2011. All children completed a protocol including questionnaires, blood collection, and spirometry conducted with a Survey Tach Spirometer (Warren E. Collins) in accordance with American Thoracic Society recommendations [8]. Written parental and participating child consent/assent was obtained. The study was approved by the Mass General Brigham Human Research Committee at Brigham and Women’s Hospital, protocol #2000-P-001130/55, and the Hospital Nacional de Niños.

### Metabolomic profiling

Metabolomic profiling was conducted using four complementary liquid chromatography tandem mass spectrometry (LC-MS) platforms as part of the TOPMed initiative [13]. Three global LC-MS methods using high resolution, accurate mass profiling measured *1*) polar and nonpolar lipids; *2*) free fatty acids, bile acids, and metabolites of intermediate polarity; and *3*) polar metabolites including amino acids, acylcarnitines, and amines. An additional targeted LC-MS profiling method measured intermediary metabolites including purines and pyrimidines, and acyl CoAs [14]. Metabolites with CV% > 25% in pooled QC samples dispersed throughout the run or those missing > 75% of samples were excluded. Remaining missing values were imputed using the k-nearest-neighbor imputation method (R package “VIM”) (https://www.jstatsoft.org/article/view/v074i07*).* Metabolites were log-10 transformed and unit-scaled. Unnamed metabolites were removed. Further details in Supplementary Methods and Figure E8.

### Metabo-endotypes

As described previously, metabo-endotypes were derived in 1151 participants with available plasma metabolomic profiling, using 589 metabolite residuals (named metabolites with age, sex and body mass index (BMI) regressed out), similarity network fusion and spectral clustering [6]. Individuals within the resulting five metabo-endotypes differed in terms of lung function metrics, with those in metabo-endotype 2 demonstrating the poorest metrics of function and those in metabo-endotype 3 the best. The metabo-endotypes were recapitulated in 911 participants from the independent Childhood Asthma Management Program [15] using the label propagation approach, and the same differences in lung function metrics were observed between the CAMP participant’s metabo-endotypes. These five metabo-endotypes form the basis of the current analysis.

### MiRNA profiling

miRNA were sequenced from serum samples. Small RNA-seq libraries were prepared using the Norgen Biotek Small RNA Library Prep Kit (Norgen Biotek, Therold, Canada) and sequenced on the Illumina NextSeq 500 platform by Norgen Biotek [16]. The ExceRpt pipeline was used for RNA-seq data QC (Figure E1) [17]. MiRNAs with < 5 mapped reads in 50% of the participants were removed from analysis. Samples with < 100,000 mapped miRNA reads were removed. Each sample yielded an average of 15.8 million total reads, of which 11.9 million per sample passed the initial quality control (QC). On average, 8.9 million reads per sample were successfully mapped to the genome, including 5.4 million miRNA sense sequences. After QC, expression data for 317 miRNAs were obtained from 1,134 subjects. DESeq2 was used to normalize the data (Figure E9) [18]. For the GACRS cohort, sequencing was performed in two batches, which could have introduced technical variability during sample preparation and handling. To assess potential batch effects on mapped read counts, guided principal component analysis (gPCA) was conducted. The analysis indicated no significant batch effect in the normalized data (*p* = 0.41) (Figure E2).

### Statistical analysis

#### Associations between metabo-enodotype membership and microRNA levels

We investigated differences in the normalized expression levels of serum microRNAs that passed QC between the five metabo-endotypes using analysis of covariance (ANCOVA) with age, sex, and BMI as covariates (Figure E1). We conducted post-hoc pairwise comparisons to identify differences between each pair of metabo-endotypes, using simultaneous tests for General Linear Hypotheses with Multiple Comparisons of Means, utilizing Tukey Contrasts [19]. For all tests, significance was determined at a level of Benjamini-Hochberg adjusted p-value < 0.05. As miRNA dat a may be highly correlated, pair-wise Pearson correlation among miRNAs that were significantly different between metabo-endotypes were calculated and a heatmap was generated using the pheatmap package in R to visualize significant correlations (*p* < 0.01). Hierarchical clustering was applied to rows and columns, followed by dendrogram analysis using cutree to define clusters.

High-confidence (functional miRNA-target interaction) target genes of the miRNAs of interest were identified using miRTarBase Version7 [20]. Pathway functional enrichment analysis for target genes was conducted using the Database for Annotation, Visualization, and Integrated Discovery 2024 [21, 22], and the KEGG database. This was followed by functional annotation clustering based on kappa statistics scores and a fuzzy clustering algorithm [21, 23].

#### Metabo-endotype classification

To determine the miRNAs of greatest interest and assess how well miRNAs could distinguish metabo-endotype membership, we built a model using the miRNAs identified as significant by ANCOVA and a LASSO approach with 10-fold cross-validation, splitting the dataset into 70:30 for training and testing. Performance was assessed by using the convex hull [24] of the area under the receiver operating characteristic curve (AUC) with confidence intervals estimated according to DeLong et al. [25].

#### Associations between metabolite and microRNA levels within metabo-endotypes

To determine if the relationships between each microRNA and each metabolite differed among individuals based on metabo-endotype membership, we employed multiple linear regression models adjusting for age, sex and BMI within a given metabo-endotype. We then tested whether there was a significant difference in effect estimates for each miRNA-metabolite pair between individuals in different metabo-endotypes using a two-sample t-test, accounting for unequal variance. The miRNA-metabolite pairs were visualized as an association network, where nodes represented miRNAs and metabolites, and edges indicated associations, and node size was proportional to the number of associations.

All analyses were conducted in R version 4.4.1.

## Results

### Study populations

Among the 1151 metabo-endotype participants, 1121 (97.4%) additionally had serum microRNAs profiling from the same blood draw. The exclusion of individuals without miRNA data did not alter the characteristics of the previously defined [6] metabo-endotypes. (Table E1); there was no significant difference in age, sex or BMI between the five groups in this analysis.

### Associations between metabo-enodotype membership and microRNA levels

**Fig. 1:**
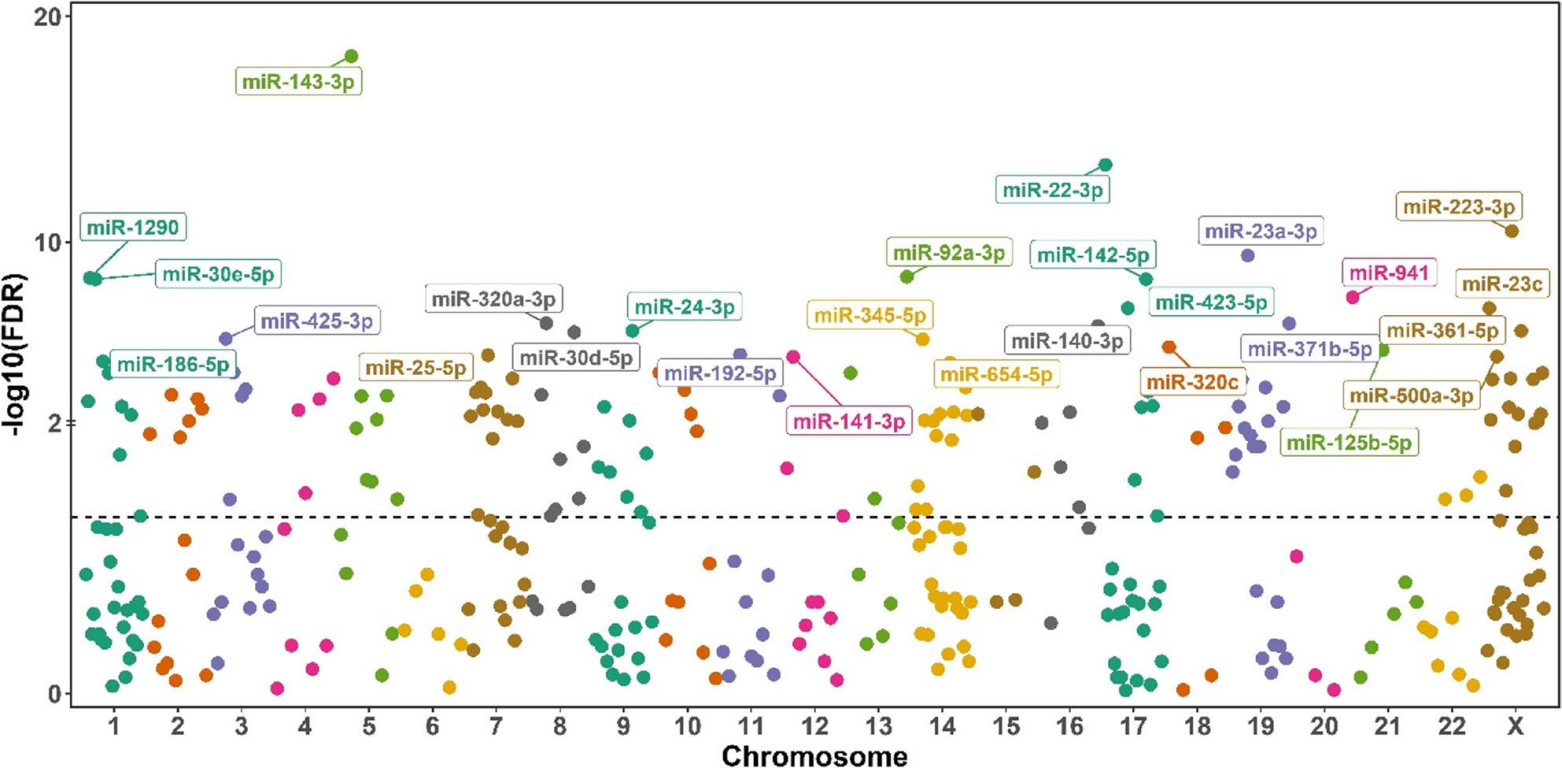
Manhattan plot demonstrating miRNAs that differ significantly across the metabo-endotypes in GACRS. MiRNAs assigned to chromosomal region using miRNA coordinate file (GFF3 file) vs 22 from MirBase, only one start position within the same chromosome is presented; miRNAs with an FDR<5x10-5 are labeled. The color of labels corresponds to chromosome number

Levels of 132 of 317 miRNA (41.6%) that passed QC differed significantly across the five metabo-endotypes (Fig. 1, Table E2) with an FDR < 0.05. These results were unchanged when we additionally adjusting for oral corticosteroid use in the last six months(yes/no) and inhaled corticosteroid use in the last six months (yes/no) to account for the reported effect of steroids on miRNA expression levels (Table E2). Of the 137 miRNA, 123 (93.2%) demonstrated high confidence target genes based on Functional MTI annotations (Table E3). Enrichment analyses of these predicted gene targets identified one significant cluster of KEGG pathways (Table 1, Figure E3), based around lymphocytes. This cluster included the pathways of Th17 cell differentiation (FDR *p* = 5 × 10^− 15^), Th1 and Th2 cell differentiation (FDR *p* = 4.37 × 10^− 8^), and Hematopoietic cell lineage (FDR *p* = 5.07 × 10^− 5^) which regulates the differentiation of immature stem cells into Hematopoietic cells including T-cells. Asthma (FDR *p* = 1.02 × 10^− 04^) and Inflammatory bowel disease (FDR *p* = 9.9 × 10^− 14^), which shares both genetic and environmental risk factors with asthma [26] were also enriched.

**Table 1:** Pathway enrichment analyses of high confidence target genes of miRNAs with differing levels across five asthma metabo-endotypes

Annotation Cluster Enrichment Score: 10.11				
Term	Gene Count (%)	Fold Enrichment	*P*-Value	FDR *p*-value
hsa04659:Th17 cell differentiation	5.017	7.211	2.58 × 10^− 17^	5.00 × 10^− 15^
hsa05321:Inflammatory bowel disease	3.846	9.185	1.02 × 10^− 15^	9.90 × 10^− 14^
hsa04658:Th1 and Th2 cell differentiation	3.344	5.643	1.35 × 10^− 09^	4.37 × 10^− 08^
hsa04640:Hematopoietic cell lineage	2.676	4.195	4.89 × 10^− 06^	5.07 × 10^− 05^
hsa05310:Asthma	1.505	7.536	1.58 × 10^− 05^	1.02 × 10^− 04^

The high confidence (functional MTI) target genes of 132 miRNAs were identified from mirTarBase database Version 7. Subsequently, these target genes were uploaded to Database for Annotation, Visualization and Integrated Discovery (DAVID) 2024, and potential biological function was analyzed using the Kyoto Encyclopedia of Genes and Genomes (KEGG) pathway enrichment analysis, followed by functional annotation clustering. Annotation Cluster Enrichment Score: the geometric mean (in -log scale) of member's p-values in a corresponding annotation cluster, is used to rank their biological significance

*FDR *False discovery rate

**Fig. 2:**
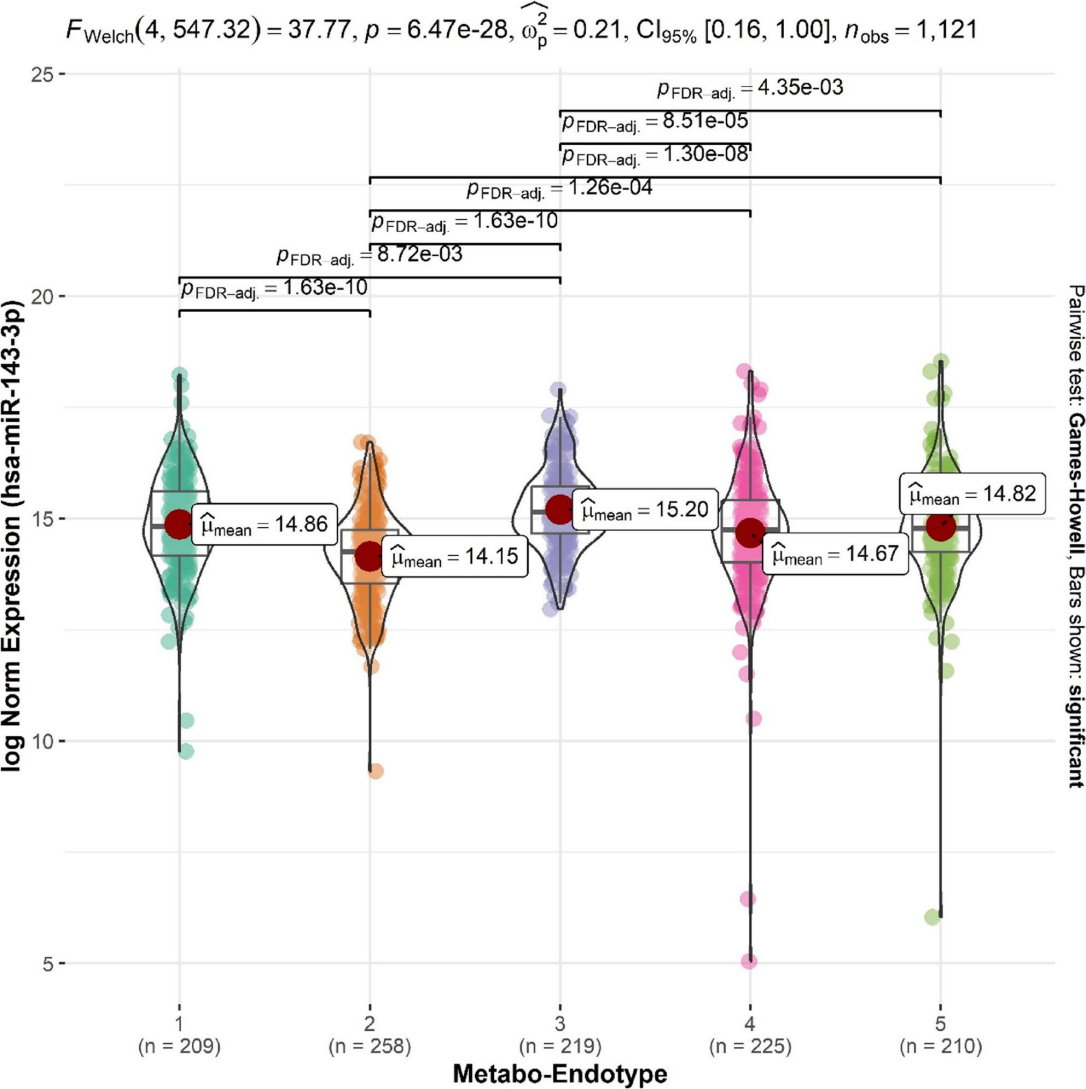
Levels of serum miR-143-3p across five metabo-endotypes. The figure shows log-normalized expression of miR-143-3p across metabo-endotypes. Welch ANOVA (F (4, 547.32) = 37.77, p = 6.47e-28) revealed significant differences across groups, with moderate effect size (η=0.21). Pairwise comparisons were conducted using the Games-Howell test, with FDR-adjusted p-values shown for significant differences. Mean expression values are annotated within each violin

The miRNA with expression levels that differed the most across the metabo-endotypes was miR-143-3p (FDR *p* = 5.7 × 10^− 19^). The Tukey *post hoc* test demonstrated that the significance of miR-143-3p was driven by pairwise significant differences between all metabo-endotypes with the exception of 4 and 5, with the lowest mean levels in metabo-endotype 2 and the highest in 3, the metabo-endotypes with the poorest and best lung function respectively [6] (Fig. 2, Figure E4). A similar pattern was observed across the top hits, of the 132 significant miRNAs, for 78 (59.1%) the highest levels were in metabo-endotype3 and the lowest in metabo-endotype 2; and for 18 (13.6%) the highest were in metabo-endotype 3 and the lowest in 2 (Table E2).

**Fig. 3:**
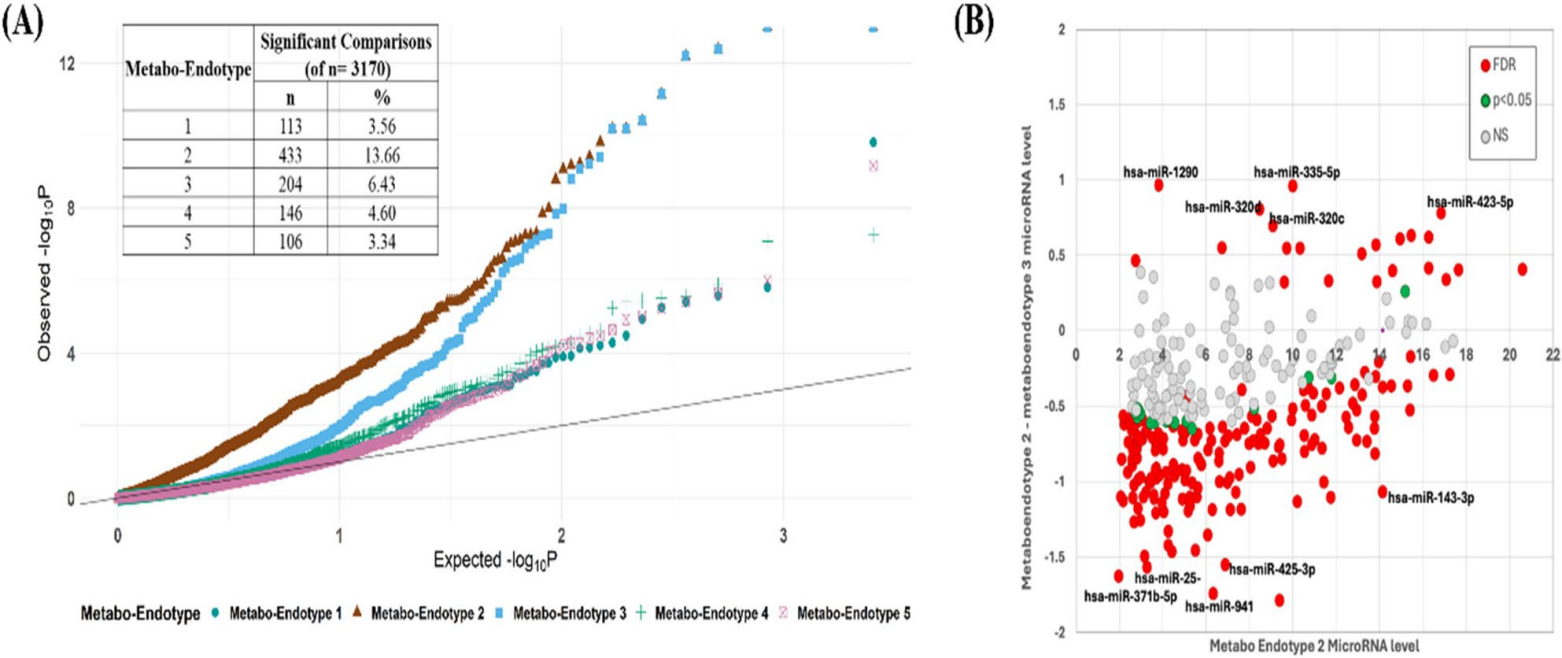
**A** QQ-plot showing the p-value distribution of contrasts that each metabo-endotype was involved. **B** Mean miroRNA levels in metaboendotype 2 plotted against the difference in mean microRNA levels between metaboendotype 2 and metaboendotype 3; colored according to the significance level of the difference

When considering all 3170 post-hoc pairwise test comparisons across the five endotypes, 501 (15.8%) were FDR significant (Table E4). Of these, 433 (86.4%) involved metabo-endotype 2, with 204 (40.7%) involving metabo-endotype 3. The comparison between metabo-endotype 2 and 3 accounted for 141 (28.2%) of the 501 FDR significant hits and QQ-plots demonstrate that there was an enrichment of highly significant findings in metabo-endotypes 2 and 3, relative to the others (Fig. 3a). Among the microRNAs with levels that differed the most between the two groups was miR-143-3p (Fig. 3b), as well as several other microRNAs previously associated with asthma and asthma phenotypes including lung function and treatment response. Enrichment analyses of the 141 microRNAs that were significantly different between metabo-endotypes 2 and 3 revealed the same pathways as the analysis based on the 132 miRNAs from the ANCOVA (126 miRNAs were common between these two sets of results), confirming that the metabo-endotypes 2 and 3 were driving the enrichment results (Table E5).

We then clustered these 141 miRNAs based on their expression levels to explore whether they were highly correlated and therefore may not be exerting independent effects. We identified five clusters of miRNAs (Figure E5) highlighting their potential cooperative as well as distinct regulatory roles. miRNA cluster2 had the highest number of shared gene targets (*n* = 323), suggesting strong functional redundancy or co-regulation among its miRNAs, and the possibility they may be targeting common biological pathways. Conversely, miRNA cluster3, had no shared gene targets, indicating miRNAs in this group may regulate unique pathways or processes independently. Clusters1, 4, and 5 show moderate shared targets (*n* = 4 to *n* = 20), implying a balance between individual specificity and shared regulatory functions. This variation in shared target genes across miRNA clusters reflects the functional diversity and complexity of miRNA-mediated gene regulation on membership of metabo-endotypes 2 and 3.

The dominance of metabo-endotypes 2 and 3 and the significance of asthma relevant miRNAs, including has-miR-143-3p, were consistent when we used logistic regression and a one-versus-the rest approach, comparing levels in individuals within a given metabo-endotype, to those in individuals in all other metabo-endotypes combined, with adjustment for age, sex, and BMI, (Table E6, Figure E6).

**Fig. 4:**
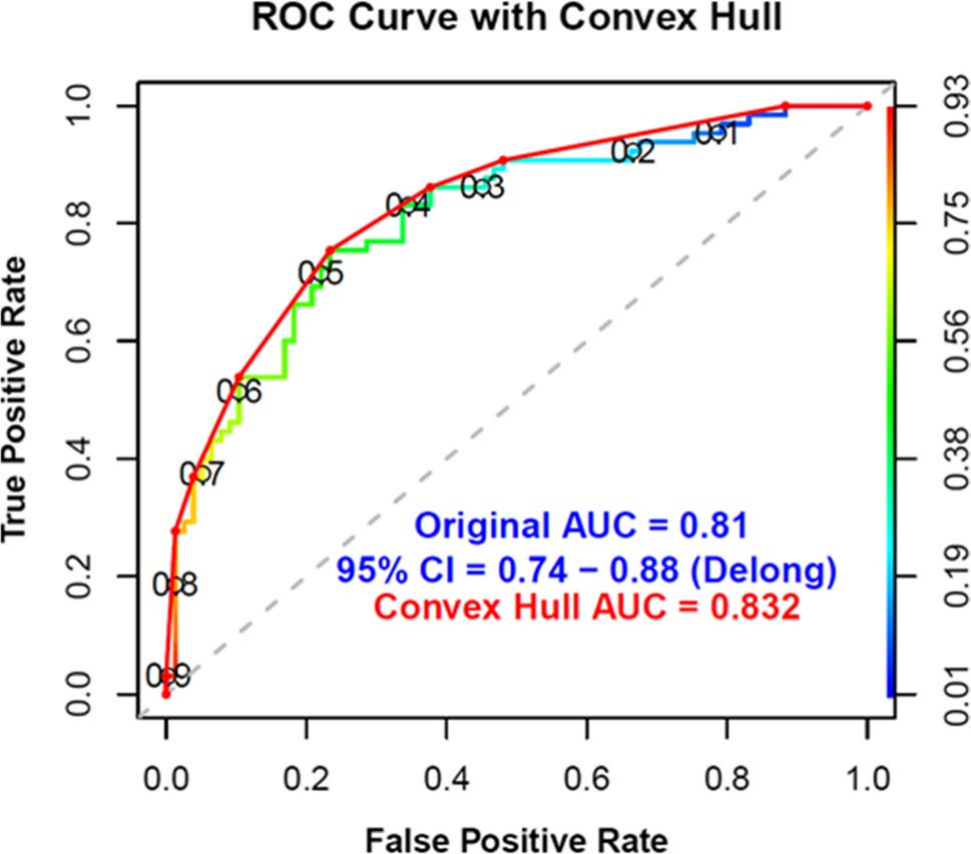
Model for Metabo-endotype Differentiation Using miRNAs. A LASSO model was built to identify miRNA most important for the discrimination of membership of meta-endotype 2 versus 3 in the GACRS cohort using 17 miRNAs. The dataset was split 70:30 for training and testing, with 10-fold cross-validation. Model performance was evaluated by the AUC, with confidence intervals estimated by DeLong’s method, applying the convex hull for optimal AUC selection

Next, a miRNA model for distinguishing between endotype 2 and endotype 3 was developed using the 141 miRNAs and LASSO regression with 10-fold cross-validation. The purpose was to identify the subset of the most biologically informative miRNAs, by removing highly correlated and redundant miRNAs, and to determine the ability of this subset to accurately classify membership. The final model was trained on 70% of the data and retained 17 miRNAs: miR-143-3p, miR-191-5p, miR-30e-5p, miR-140-3p, miR-223-3p, miR-320a-3p, miR-92a-3p, miR-122-5p, miR-23a-3p, miR-484, miR-125b-5p, miR-335-5p, miR-371b-5p, miR-25-5p, miR-23c, miR-320e, and miR-1290. When tested on the remaining 30% of participants, this model achieved an AUC of 81% (CI: 73% − 88%) (Fig. 4). Seventeen miRNAs identified by the LASSO model were predicted to target 336 genes, which were used to construct a protein–protein interaction (PPI) network using STRINGdb (Figure E7A). The resulting network revealed extensive interconnectivity among target genes, suggesting coordinated regulatory activity. KEGG pathway enrichment analysis of these genes demonstrated significant enrichment in immune and inflammatory signaling pathways, including inflammatory bowel disease, Th17 cell differentiation, and Th1/Th2 cell differentiation (Figure E7B). These findings highlight the biological relevance of the 17-miRNA signature and its potential role in regulating immune-mediated mechanisms underlying disease endotypes.

#### Comparisons of metabolite~microRNA relationships between individuals in metabo-endotype 2 and those in metabo-endotype 3

Finally, we sought to compare the relationships between each microRNA and each metabolite in individuals in metabo-endotype 2 versus metabo-endotype 3 (Total association between 317 miRNAsX589 metabolites = 1,86,713). There was a significantly different microRNA ~ metabolite effect estimate in the children in metabo-endotype 2 (*n* = 258) as compared to those in metabo-endotype 3 (*n* = 219) for 12,626 microRNA ~ metabolite pairs based on a nominal *p* < 0.05 threshold. None were FDR significant. These pairs whose effect estimates differed with nominal significance included 528 unique metabolites and 316 unique miRNAs. Most of these microRNA ~ metabolite associations were in different directions of effect between individuals in metabo-endotype 2 versus 3, however 778 were in the same direction but with different magnitudes of effect (Table E7).

**Fig. 5:**
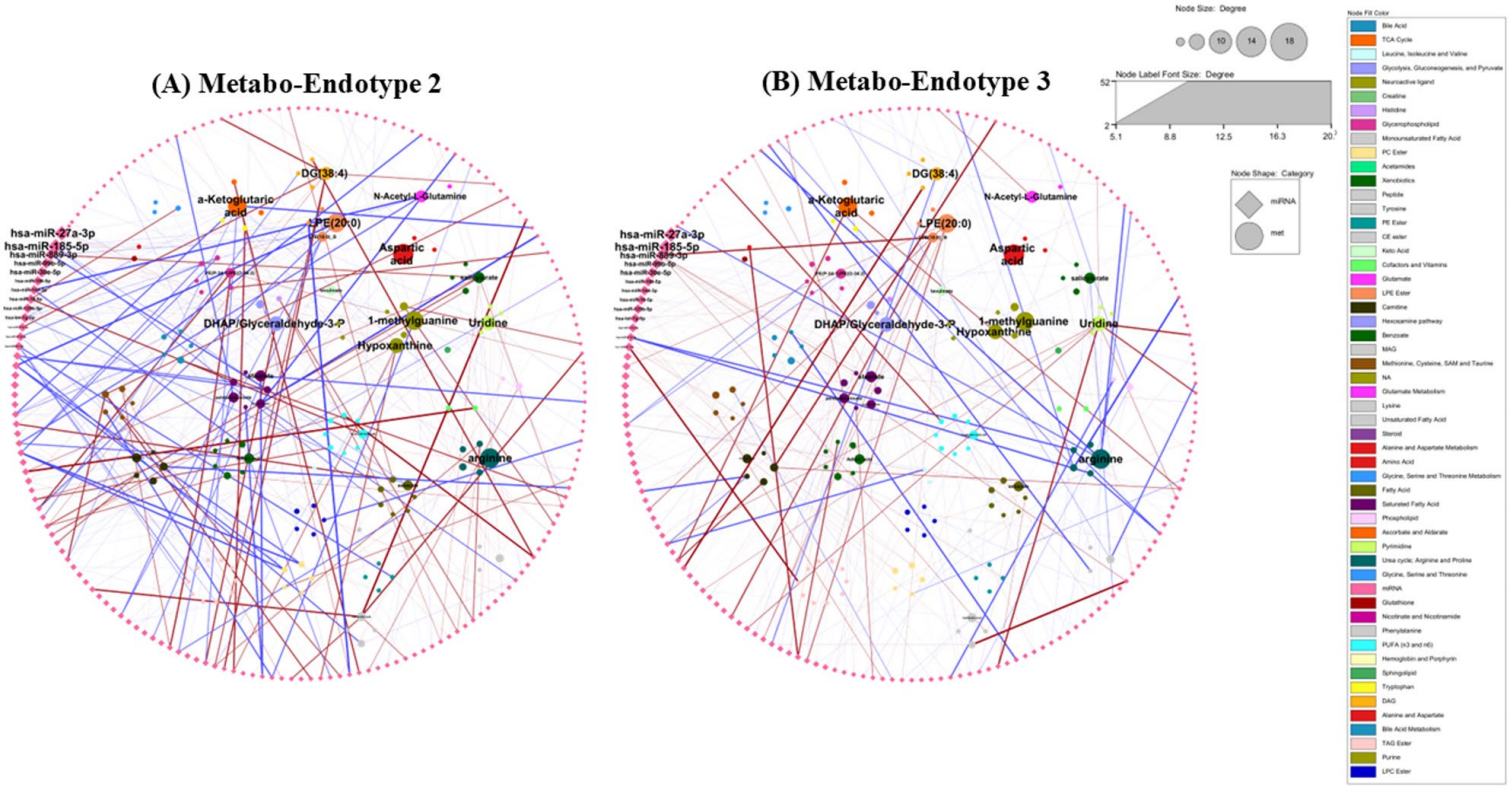
MicroRNA~Metabolite associations in individuals in (**A**) metabo-endotype 2 and (**B**) metabo-endotype 3, for 421 associations that differed in direction and magnitude between the two metabo-endotypes at p<0.001 significance level

Figure 5 visualizes the differences in associations between metabo-endotypes 2 and 3. These included differences between miRNAs with several known asthma metabolites such as eicosinoids, aspartic acid, arginine, 1-methylguanine, a-Ketoglutaric acid, LPE(20:0), DHAP/Glyceraldehyde-3-P, hypoxanthine, uridine, and DG(38:4). These metabolites form hubs within the network plots and were key drivers of the metabolomic differences between metabo-endotypes in the original manuscript, suggesting that miRNAs may be playing an important role in the formation of clinically distinct asthma metabo-endotypes.

## Discussion

The identification of asthma ‘endotypes’ that share biological mechanistic features, is crucial to the development of targeted prevention and management strategies for this common chronic condition. We previously derived and validated five asthma endotypes with differing severity metrics using metabolomic data [6]. MiRNAs have rapidly gained recognition as key natural regulators of global gene expression, with numerous studies showing that miRNAs are influenced by metabolic stimuli like nutrients, hormones, and cytokines. In turn, miRNAs can regulate metabolism, forming a bidirectional relationship that, when disrupted, can disturb metabolic homeostasis [3, 27]. Here, we aimed to determine whether serum miRNA levels differ between individuals in our five metabo-endotypes, to reveal novel insights into their biology and to decipher the relationships between miRNAs and the key metabolites driving the formation of our endotypes with a view to explore potential post-transcriptional mechanisms.

Across the five metabo-endotypes, there was a significant difference in the circulating levels of more than 40% of the measured microRNAs, suggesting an overall difference in the regulation of gene expression between the endotypes. The most significant difference was for miR-143-3p, which targets TGF-β1, CDK4, Cyclin D1, and which has been implicated in asthma through suppression of the proliferation and deposition of extracellular membrane proteins in TGF-β1-mediated airway smooth muscle cells [28–30]. However, there is contradictory evidence regarding its direction of effect and relationship to severity, with some studies reporting that it can predict exacerbations [31]. In contrast, we observed the lowest levels in our most severe endotype, and the highest in our least severe. The pattern of lowest levels in metabo-endotype 2 and highest in 3 was seen for the majority of the top hits, most of which have similarly been previously associated with asthma, including miR-22-3p [32], miR-223-3p [33], miR-23a-3p [33] and miR-1290 [34] among others. Overall, differences in miRNA levels across the endotypes were primarily driven by metabo-endotypes 2 and 3, which represent the “most” and “least” severe asthma cases, respectively, distinguished by differences in airflow obstruction and corticosteroid and β2-agonist use [6]. It has been shown that treatments for asthma including steroid can influence the expression of microRNAs including miR-143-3p [35], however a sensitivity analysis demonstrated that our results were unaffected when we adjusted for ICS and OSC use.

Enrichment analysis of the predicted target genes for all miRNAs that differed significantly across the metabo-endotyes suggested an enrichment of genes involved in asthma and asthma-relevant processes relating to T cell differentiation, the hematopoietic cell lineage pathway and inflammatory bowel disease. A growing a growing number of studies have reported an association between asthma and IBD [36–40]. This association is attributed to several mechanisms including through the gut-lung axis and shared microbial and environmental risk factors [39]. There is also compelling evidence that they share a genetic architecture [40]. Studies have reported several risk genes associated with susceptibility to both conditions including *SMAD3*, *GSDMB*, *ORMDL3* [38, 40], all of which were targets of the miRNA included in our enrichment analysis. As such, we believe this result is due to the genetic concordance of the two conditions. These predicted target genes included those associated with both the onset of childhood asthma, such as *ORMDL3* [41], *GSDMB* [42], *IL33* [43] and *ZPBP2* [44], and those previously associated with asthma exacerbations and severity including *CDHR3* [45], *RANBP6* [46] and *IL13* [47]. One of the main metabolomic differentiators between the metabo-endotypes was levels of polyunsaturated fatty acids. A key step in the metabolism of these inflammatory mediators is catalyzed by a desaturation encoded by *FADS2*, which was also among the target genes and the expression of which has been previously associated with asthma phenotypes [48]. Similarly, 9,10-diHOME levels were a primary driver of metabo-endotype membership [6], 9,10-diHOME is hydrolyzed by soluble epoxide hydrolase which is encoded by *EPHX2*, another target gene. In this way we can hypothesize that these miRNAs might be driving metabo-endotype membership through the targeting of asthma related susceptibility loci, and the subsequent influences on metabolite levels.

The enrichment for genes of hematopoietic cell lineage and the Th1, Th2, and Th17 cell differentiation pathways suggests that the regulatory influence of these miRNAs extends across multiple immune pathways central to asthma pathophysiology. These findings are consistent with the well-established role of Th2-mediated eosinophilic inflammation in severe asthma [49, 50]. Intriguingly, individuals in endotypes 2 and 3 had the highest and lowest eosinophil counts respectively across all participants (*p* = 0.009) [6]. The involvement of Th17 differentiation points to a potential Th2/Th17 overlap, contributing to the increased inflammation and treatment resistance observed in the severe endotype 2 [49, 50]. While the presence of Th1 pathway enrichment, though typically associated with non-eosinophilic inflammation [51], suggests a more complex immune regulation between endotypes. Furthermore, the hematopoietic cell lineage pathway’s involvement underscores the role of these miRNAs in regulating the differentiation and activation of key immune cells, such as eosinophils and T cells [52]. These findings highlight the potentially multifaceted roles of miRNAs in modulating immune responses in asthma. Furthermore, using LASSO regression with 10-fold cross-validation, we found that 17 key miRNAs achieved an AUC of > 80% in the validation set to distinguish membership of metabo-endotype 2 versus 3, highlighting their potential role in the molecular differentiation of these endotypes. Of these 17, miR-143-3p, miR-140-3p, miR-223-3p, miR-320a-3p, miR-92a-3p, miR-122-5p, miR-125b-5p, miR-335-5p and miR-1290 have been directly implicated in asthma and asthma phenotypes [29, 33, 53, 54], with the remainder implicated in related processes such as immunity, or other respiratory conditions including chronic obstructive pulmonary disease. However, it is important to note that miRNAs can be highly correlated. The fact that the LASSO model identified only 17 miRNAs, as well as our clustering of the miRNAs suggests that although a large proportion of miRNAs were identified in our primary analyses, not all may have biological relevance, and further work, including potentially functional follow up, is required to identify the driver miRNAs of most importance.

We subsequently aimed to determine whether the association between a given microRNA ~ metabolite pair differed between individuals classified into metabo-endotype 2 versus those in metabo-endotype 3. We determined that for a subset of microRNAs, their associations with specific metabolites varied significantly between these two metabo-endotypes, although we note only nominal significance was achieved. This difference suggests that the regulatory roles of microRNAs in metabolic pathways may manifest differently in metabo-endotypes 2 and 3, potentially elucidating the observed differences in phenotypic manifestations of asthma. For instance, miR-143-3p showed a nominally significant positive correlation with hippurate, a gut microbiota-derived polyphenol metabolite previously associated with asthma [5], in individuals from metabo-endotype 2, while a negative correlation was observed in those from metabo-endotype 3. Similarly, miR-503-5p, miR-654-3p, miR-4738-3p, miR-654-5p, miR-340-5p, miR-374b-5p, miR-335-5p, miR-493-3p, miR-199a-3pXmiR-199b-3p, miR-136-3p, and miR-151b exhibited negative correlations with 12,13-diHOME in metabo-endotype 2 but positive correlations in metabo-endotype 3. This differential regulatory mechanism highlights the complexity of post-transcriptional control and implies that differences in microRNA expression and their metabolic associations may contribute to the clinical heterogeneity and severity of asthma. Visualization of the differences in miRNA-metabolite relationships between metabo-endotypes 2 and 3 identified several hub metabolite groups, including eicosanoids, tri-gylcerides and ceramides and all of which have been previously implicated in asthma phenotypes, and many of which were identified and key driver metabolites in the original publication, again providing evidence that that metabo-endotype membership may in part by driven by differential post transcriptional regulation by miRNAs. However, it is important to note that the relationship between metabolites and miRNA is bidirectional, while miRNA can regulate cellular metabolism, metabolic stimuli have also been shown to alter miRNA expression [55–57]. In this current study, miRNA and metabolites were measured contemporaneously and therefore we cannot determine whether miRNAs are influencing metabolite levels, or if the reverse is true. As we only had metabolites and miRNAs at a single timepoint, we could also not assess the temporal stability of these associations. In fact, a recent review concluded that there are very few studies exploring the stability of miRNA profiles over time and those that do exist are limited by very small sample sizes and a small number of miRNA [58]. Therefore, more work is needed to determine the temporal stability of both miRNAs and miRNA ~ metabolite associations, and whether such associations are likely to change with disease progression or treatment.

There were several other limitations to these analyses. Although our metabo-endotypes were validated in our previous publication, we do not have a suitable validation cohort with contemporaneous measures of miRNA to validate these current findings. We recognize that we do not have full coverage of the miRNAome. Therefore, there are likely additional differences between the metabo-endoypes and pertinent miRNA ~ metabolite relationships that could not be captured by our analyses. We demonstrated that there is high correlation between many of our miRNA of interest, and further work is required to identify the most biologically relevant. Although we note that of those miRNAs identified in our LASSO model, which addresses the issue of correlation, many have been previously associated with asthma, a key limitation of this study is the absence of mRNA expression data and the lack of experimental validation for the predicted miRNA targets. We also note that the 70:30 test and discovery approach used for our classification model cannot completely eliminate the risk of overfitting, given the miRNAs were preselected from the same population. Only nominal significance was achieved in the miRNA ~ metabolite analysis, although this could be a function of power. Finally, although we adjust for sex, age and BMI in these analyses, there may be additional measured and unmeasured confounders that are not accounted for. In particular although we adjusted for ICS and OSC use in a sensitivity analyses, we did not have detailed information on frequency or dosage for these or other asthma medications. It should also be noted that these findings may not be generalizable to populations with markedly different therapeutic regimens. Despite these limitations, this unique study is the first to demonstrate that miRNA levels differ between metabo-endotypes of asthma and can help to explain their genetic and biological underpinnings. It leverages a genetically homogenous and deeply phenotyped population of children with mild to moderate asthma, and further work is necessary to determine the generalizability of these findings to other populations.

In conclusion, this study suggests the interplay between miRNAs and metabolites is key to the clinical presentation of asthma and its varying phenotypes, and that biologically and clinically different asthma metabo-endotypes may demonstrate differential miRNA regulated gene expression. Increasing understanding of these relationships, including direction of effect and the underlying causes of changes in the driver omic can help to identify novel targets for the management and treatment of this common chronic condition. Further investigation into the functional implications of these miRNA ~ metabolite relationships could offer insights into the biological processes driving these specific asthma endotypes and potentially guide more personalized therapeutic strategies.

## Supplementary Information

Supplementary Material 1.

Supplementary Material 2.

Supplementary Material 3.

Supplementary Material 4.

Supplementary Material 5.

## Data Availability

RS and RSK have full access to all the data in the study and take responsibility for the integrity of the data analysis. The metabolomic data were generated as part of the NHLBI Trans-Omics for Precision Medicine Initiative (TOPMed). These data are available to the scientific community via NIH-designated repositories according to. Full details can be found at https://www.nhlbiwgs.org/topmed-data-access-scientific-community. TOPMed Accession # phs000988. The miRNA data can be accessed via https://www.ncbi.nlm.nih.gov/geo/query/acc.cgi?acc=GSE244573.

## Code availability

All statistical analyses were conducted using freely available packages in R version 4.0.0; all such packages are stated and referenced in the methods and supplementary methods.
