# Supplementary material for: Differences in microRNA levels across metabo-endotypes reveal novel insights into asthma heterogeneity
Source: Respir Res. 2026 Jan 7;27:40. doi: 10.1186/s12931-025-03452-x (PMC12869918; doi:10.1186/s12931-025-03452-x)
Supplement: Supplementary file 5 — Supplementary Material 5. [file 12931_2025_3452_MOESM5_ESM.docx]

**ONLINE DATA SUPPLEMENT**

**MicroRNA Differences Across Metabo-endotypes Reveal Post Transcriptional Regulation**

Rinku Sharma*, Rachel Kelly*, Kevin Mendez, Qingwen Chen, Julian Hecker, Sofina Begum, Clary Clish, Juan C. Celedón, Kelan G. Tantisira, Scott T. Weiss, Jessica Lasky-Su**, Michael McGeachie** on behalf of the NHLBI Trans-Omics for Precision Medicine (TOPMed) Consortium

***Co-first authors**

****Co-last authors**

**Supplementary Methods**

**The Genetics of Asthma in Costa Rica Study (GACRS)**

As described previously^1^, GACRS ^2^ recruited 1,165 children with asthma, aged 6-14 years, from 140 school across the Central Valley of Costa Rica between February 2001 and August 2008. Children were eligible for the study if they had asthma, as defined by physician-diagnosis and ≥2 respiratory symptoms or asthma attacks in the prior year, and a high probability of having ≥6 great-grandparents born in the Central Valley of Costa Rica ^2^. At enrollment, all children completed a protocol including spirometry, questionnaires, and collection of blood samples. The questionnaire was a version of the one used in the Collaborative Study on the Genetics of Asthma translated into Spanish ^3^, and included information on medication use, hospitalizations, and physician’s diagnoses of hayfever and eczema/atopic dermatitis. Spirometry was conducted with a Survey Tach Spirometer (Warren E. Collins; Braintree, MA) in accordance with American Thoracic Society recommendations ^2^. Written parental and participating child consent was obtained. The study was approved by the Partners Human Research Committee at Brigham and Women’s Hospital (Boston, USA); Protocol#: 2000-P-001130/55, and the Hospital Nacional de Niños (San José, Costa Rica).

**LC-MS method descriptions**

Methods have been described in detail previously^1^.

**C8-pos: Reversed-phase C8 chromatography/positive ion mode MS detection to measure polar and non-polar plasma lipids (Broad Institute)** using an LC-MS system comprised of a Shimadzu Nexera X2 U-HPLC (Shimadzu Corp.) coupled to an Exactive Plus orbitrap mass spectrometer (Thermo Fisher Scientific). Plasma samples (10 µL) were extracted using 190 µL of isopropanol containing 1,2-didodecanoyl-sn-glycero-3-phosphocholine (Avanti Polar Lipids) as an internal standard. After centrifugation, supernatants were injected directly onto a 100 x 2.1 mm, 1.7 µm ACQUITY BEH C8 column (Waters). The column was eluted isocratically with 80% mobile phase A (95:5:0.1 vol/vol/vol 10mM ammonium acetate/methanol/formic acid) for 1 minute followed by a linear gradient to 80% mobile-phase B (99.9:0.1 vol/vol methanol/formic acid) over 2 minutes, a linear gradient to 100% mobile phase B over 7 minutes, then 3 minutes at 100% mobile-phase B. MS analyses were carried out using electrospray ionization in the positive ion mode using full scan analysis over 200–1100 m/z at 70,000 resolution and 3 Hz data acquisition rate. Other MS settings were: sheath gas 50, in source CID 5 eV, sweep gas 5, spray voltage 3 kV, capillary temperature 300°C, S-lens RF 60, heater temperature 300°C, microscans 1, automatic gain control target 1e6, and maximum ion time 100 ms. Raw data were processed using TraceFinder software (Thermo Fisher Scientific) for targeted peak integration and manual review of a subset of identified lipids and using Progenesis QI (Nonlinear Dynamics) for peak detection and integration of both lipids of known identify and unknowns. Lipid identities were determined based on comparison to reference plasma extracts and are denoted by total number of carbons in the lipid acyl chain(s) and total number of double bonds in the lipid acyl chain(s).

**C18-neg: Reversed-phase C18 chromatography/negative ion mode MS detection to measure free fatty acids, bile acids, and metabolites of intermediate polarity (Broad Institute)** using an LC-MS system comprised of a Shimadzu Nexera X2 U-HPLC (Shimadzu Corp.) coupled to a Q Exactive hybrid quadrupole orbitrap mass spectrometer (Thermo Fisher Scientific). Plasma samples (30 µL) were extracted using 90 uL of methanol containing 15R-15-methyl-PGA_2_, 15R-15-methyl-PGF_2alpha_, 15S-15-methyl-PGD_2_, 15S-15-methyl-PGE_1_, and 15S-15-methyl-PGE_2_ (Cayman Chemical Co.) internal standards and centrifuged (10 min, 9,000 x g, 4°C). The samples were injected onto a 150 x 2 mm ACQUITY BEH C18 column (Waters). The column was eluted isocratically at a flow rate of 400 µL/min with 60% mobile phase A (0.1% formic acid in water) for 4 minutes followed by a linear gradient to 100% mobile phase B (acetonitrile with 0.1% acetic acid) over 8 minutes. MS analyses were carried out in the negative ion mode using electrospray ionization, full scan MS acquisition over 200-550 m/z, and a resolution setting of 70,000. Other MS settings were: sheath gas 45, sweep gas 5, spray voltage -3.5 kV, capillary temperature 320°C, S-lens RF 60, heater temperature 300°C, microscans 1, automatic gain control target 1e6, and maximum ion time 250 ms. Raw data were processed using TraceFinder software (Thermo Fisher Scientific) for targeted peak integration and manual review of a subset of identified metabolites and using Progenesis QI (Nonlinear Dynamics) for peak detection and integration of both metabolites of known identify and unknowns. Metabolite identities were confirmed using authentic reference standards.

**HILIC-pos: Hydrophilic interaction liquid chromatography/positive ion mode MS detection to measure water-soluble polar metabolites (Broad Institute)** using an LC-MS system comprised of a Shimadzu Nexera X2 U-HPLC (Shimadzu Corp.) coupled to a Q Exactive hybrid quadrupole orbitrap mass spectrometer (Thermo Fisher Scientific). Plasma samples (10 µL) were prepared via protein precipitation with the addition of nine volumes of 74.9:24.9:0.2 v/v/v acetonitrile/methanol/formic acid containing stable isotope-labeled internal standards (valine-d8, Sigma-Aldrich; St. Louis, MO; and phenylalanine-d8, Cambridge Isotope Laboratories; Andover, MA). The samples were centrifuged (10 min, 9,000 x g, 4°C), and the supernatants were injected directly onto a 150 x 2 mm, 3 µm Atlantis HILIC column (Waters). The column was eluted isocratically at a flow rate of 250 µL/min with 5% mobile phase A (10 mM ammonium formate and 0.1% formic acid in water) for 0.5 minute followed by a linear gradient to 40% mobile phase B (acetonitrile with 0.1% formic acid) over 10 minutes. MS analyses were carried out using electrospray ionization in the positive ion mode using full scan analysis over 70-800 m/z at 70,000 resolution and 3 Hz data acquisition rate. Other MS settings were: sheath gas 40, sweep gas 2, spray voltage 3.5 kV, capillary temperature 350°C, S-lens RF 40, heater temperature 300°C, microscans 1, automatic gain control target 1e6, and maximum ion time 250 ms. Raw data were processed using TraceFinder software (Thermo Fisher Scientific) for targeted peak integration and manual review of a subset of identified metabolites and using Progenesis QI (Nonlinear Dynamics) for peak detection and integration of both metabolites of known identify and unknowns. Metabolite identities were confirmed using authentic reference standards.

**Amide-neg: Targeted negative ion mode analysis of central metabolites (BIDMC).** Central metabolites including sugars, sugar phosphates, organic acids, purine, and pyrimidines, were extracted from 30 µL of plasma using acetonitrile and methanol and separated using a 100 x 2.1 mm XBridge Amide column (Waters). A high sensitivity Agilent 6490 QQQ MS (Agilent) was used to profile metabolites in the negative ion mode via multiple reaction monitoring (MRM) scanning. MRM parameters for approximately 200 metabolites were previously optimized by infusing authentic reference standards. Raw data were processed using MassHunter Quantitative Analysis Software (Agilent).

**QC prior to and during data acquisition.**

The analytical performance of the LC-MS systems and the quality of the metabolomics data were assured using several strategies^1^. Before analyses of the study samples were initiated, reference plasma extracts and mixtures of synthetic reference standards containing up to ~150 metabolites each were analyzed to assure reproducibility of chromatographic retention times, quality of chromatographic peak shapes, and the sensitivity of the MS system. On a daily basis throughout the analytical run, internal standard signals were monitored in each sample to ensure that each injected properly and to monitor MS sensitivity. Generally, outliers (failed samples) were identified when internal standard levels were more than 2 standard deviations from the mean. Outlier samples were selected for reanalysis, using the same sample extract if the outlier was flagged within a day of its extraction, otherwise a fresh extract was prepared and analyzed**.** In addition, pairs of pooled reference samples (“PREFA” and “PREFB”) were inserted in the analysis queue at intervals of approximately 20 study samples for determination of reproducibility and data standardization and to monitor and adjust for batch effects. The pooled reference sample was created using small aliquots from each study sample at the time the samples are aliquoted for the four profiling methods. One pooled reference sample from each pair (either PREFA or PREFB) was used to standardize data across the run and between batches using “nearest-neighbor” scaling while a second pooled reference from each pair was used to calculate coefficients of variation (CVs) for every metabolite and unknown measured throughout the analysis^4^. Metabolite signals in PREF samples were also evaluated daily to assure LC retention times and peak shapes. If the pooled reference sample data showed a loss of analytical performance, the queue was stopped until the problem was corrected and the analysis queue was restarted from the last point at which data quality was acceptable.

**Non targeted data processing**

Raw LC-MS data were acquired to the data acquisition computer interfaced to each LC-MS system and then stored on a robust and redundant file storage system (Isilon Systems) accessed via the Broad’s internal network. For data processing, the platform is equipped with >10 powerful workstations configured with multi-core XEON processors, >32 GB of RAM and 2 TB of fast storage (RAID 0 arrays of four drives or NVME ssd). Targeted data processing of known metabolites was achieved using TraceFinder software (Thermo Fisher Scientific). Identities of >600 plasma metabolites have been confirmed using authentic reference standards (MSI Level 1 ID^5^) and mixtures of reference standards and reference samples were included in each analysis queue to confirm IDs in every dataset. High resolution, nontargeted data were processed using Progenesis QI software (Nonlinear Dynamics) to detect peaks, perform chromatographic retention time alignment, and integrate peak areas. Metabolites of confirmed identity were then annotated in the dataset and unknowns are “tagged” using their measured mass to charge ratio (m/z) and retention time (RT). A significant challenge for large-scale, nontargeted metabolomics studies is accurate “alignment” on unknowns among batches of samples acquired over time. This challenge arises because of the large number of nontargeted features detected in every dataset and the occurrence of minor deviations in measured m/z and RT for each peak as a function of differences in instrument calibration and LC column performance over time. The Broad lab has developed an innovative feature alignment algorithm that overcomes this challenge. The software tool is deployed as a web app and uses a unique approach to detect landmark features and non-parametric retention time scaling to accurately match unknowns between datasets. The output from this workflow is a table of concatenated data from each of the methods, expressed as individual samples in columns and metabolite abundances in rows.

**Data Levels**

In this analysis only Broad defined level 3 quality data was used. i.e., data aggregated in features. That is, redundant ion features were filtered out of the non-targeted datasets. The electrospray ionization process used in LC-MS can generate multiple ion features from a single analyte. Though these methods are aimed at maximizing the relative abundance of [M+H]+ and [M-H]- ions, may also detect lower abundance ion adducts and source fragments. The Broad lab has developed a Python-based web app that can process tabularized “normalized data” and annotates clusters of highly correlated, co-eluting LC-MS peaks. This method computes Spearman correlation coefficients among all LC-MS peaks that coelute within 0.025 minutes of one another. “Cliques” of highly correlated features are then identified based on a Spearman coefficient threshold of 0.8. The algorithm determines whether an [M+H]+ ion (positive ion mode data) or an [M-H]- ion (negative ion mode data) can be identified using mass differences among ions in each clique and, if so, selects that feature as the major ion. Otherwise, the most abundant ion was is selected as the major ion. Minor ions (i.e. redundant ions from each clique) are removed from the dataset.

The majority of metabolites are MSI level 1 validation with authentic standards. The Amide-Neg method was a targeted acquisition and, as such, the transitions required for measuring all compounds would have required authentic standards by default.

**Data Processing post data acquisition**

Pooled plasma QC samples were included throughout the assay after intervals of approximately 20 study samples. Metabolites with CV% > 25% or missing > 75% were excluded. Remaining missing values were imputed using the k-nearest-neighbor imputation method (R package "VIM")^6^. Cluster number k was designated as 3 as recommended ^7^. We examined the position of pooled plasma QC samples on principal component analysis (PCA) plots, to ensure they formed a tight cluster in the first two principal components (PCs). All metabolites were log-10 transformed and unit-scaled, and distribution of PCs according to demographic variables were examined to ensure these were not driving the PCs. Unnamed metabolites were removed.

**Supplementary Tables**

**Table E1: Characteristics of the metabo-endotypes when based on the total population and when based on those with both metabolomic and miRNA data**

| **Endotype** | **1** | | **2 "Most-severe"** | | **3 "Least-Severe"** | | **4** | | **5** | |
| --- | --- | --- | --- | --- | --- | --- | --- | --- | --- | --- |
| **Characteristic** | **Metabolomics** | **Metabolomics+miRNA** | **Metabolomics** | **Metabolomics+miRNA** | **Metabolomics** | **Metabolomics+miRNA** | **Metabolomics** | **Metabolomics+miRNA** | **Metabolomics** | **Metabolomics+miRNA** |
| **N** | 213 | 209 | 270 | 258 | 222 | 219 | 232 | 225 | 214 | 210 |
| **SEX: Male** | 125 (59%) | 123 (59%) | 160 (59%) | 153 (59%) | 136 (61%) | 134 (61%) | 133 (57%) | 131 (58%) | 128 (60%) | 124 (59%) |
| **Age** | 9.08  (7.75, 10.64) | 9.14  (7.77, 10.64) | 8.94 (7.81, 10.50) | 8.90 (7.82, 10.50) | 9.26 (7.68, 10.72) | 9.26 (7.66, 10.74) | 8.78 (7.40, 10.65) | 8.75 (7.39, 10.70) | 8.99 (7.80, 10.79) | 9.03 (7.81, 10.81) |
| **BMI** | 17.3  (15.6, 20.5) | 17.3  (15.6, 20.5) | 17.4  (15.5, 20.5) | 17.5  (15.6, 20.6) | 17.3  (15.5, 20.5) | 17.3  (15.5, 20.6) | 17.2 (15.6, 19.7) | 17.2 (15.6, 19.6) | 16.6 (15.6, 20.0) | 16.7 (15.6, 20.0) |

*The most- and least-severe metabo-endotypes were based on lung function metrics as described in PMID: 34767496*

*Metabolome refers to number of participants in the meta-endotypes as defined in this paper*

*miRNA refers to the number of people from the metabo-endotypes who also had miRNA levels measured concurrently*

**Table E2: ANCOVA results demonstrating differences in MiRNAs levels across five metabo-endotypes in GACRS with additional adjustment for ICS use in the last six months (yes/no) and OCS use in the last six months (yes/no)** *Age, sex, BMI, OCS use and ICS use included as covariates* *MeanSq: the sum of squares divided by the degrees of freedom. The highest and lowest mean level across the endotypes are shaded in green and in orange respectively*

*See excel file*

**Table E3. High confidence target genes of ANCOVA significant miRNAs.**

*See excel file*

**Table E4: Posthoc pairwise comparison of miRNA expression levels between each metabo-endotype pair using simultaneous tests for General Linear Hypotheses with Multiple Comparisons of Means**

*See excel file*

**Table E5**: **Pathway** **Enrichment Analyses of high confidence target genes of miRNAs that were significantly different between metabo-endotypes 2 and 3 based on the post-hoc test**

| **Term** | **Count** | **%** | **P-Value** | **Fold Enrichment** | **FDR** |
| --- | --- | --- | --- | --- | --- |
| hsa04659:Th17 cell differentiation | 30 | 4.93 | 3.12x10^-17^ | 7.16 | 8.98x10^-15^ |
| hsa05321:Inflammatory bowel disease | 23 | 3.78 | 1.16x10^-15^ | 9.12 | 3.20x10^-13^ |
| hsa04658:Th1 and Th2 cell differentiation | 20 | 3.29 | 1.52x10^-09^ | 5.60 | 4.38x10^-07^ |
| hsa04640:Hematopoietic cell lineage | 16 | 2.63 | 5.32x10^-06^ | 4.17 | 1.53x10^-03^ |
| hsa05310:Asthma | 9 | 1.48 | 1.66x10^-05^ | 7.48 | 4.78x10^-03^ |

*The high confidence (functional MTI) target genes of 132 miRNAs were identified from mirTarBase database Version 7 (Supplementary Table E7). Subsequently, uploaded these target genes to Database for Annotation, Visualization and Integrated Discovery (DAVID) 2024, and potential biological functions was analyzed using the Kyoto Encyclopedia of Genes and Genomes (KEGG) pathway enrichment analysis, followed by functional annotation clustering.*

**Table E6: Logistic regression results using a one versus the rest approach comparing microRNA levels across metabo-endotypes**

| **Metabo-endotype** | **N participants in metabo-endotype** | **N participants in all other metabo-endotypes** | **p<0.05** | | **p<FDR** | |
| --- | --- | --- | --- | --- | --- | --- |
|  |  |  | **n mRNAs** | **%** | **n miRNAs** | **%** |
| **1** | **209** | **912** | **7** | **2.2%** | **0** |  |
| **2** | **258** | **863** | **217** | **68.5%** | **201** | **63.4%** |
| **3** | **219** | **902** | **107** | **33.8%** | **57** | **18.0%** |
| **4** | **225** | **896** | **35** | **11.0%** | **0** |  |
| **5** | **210** | **911** | **8** | **2.5%** | **0** |  |

*Adjusting for age, sex and BMI*

**Table E7: 297 MiRNA~metabolite associations that differed between individuals in metabo-endotype 2 and metabo-endotype 3**

*See excel file*

**Supplementary Figures**

**Figure E1: Study Schematic**


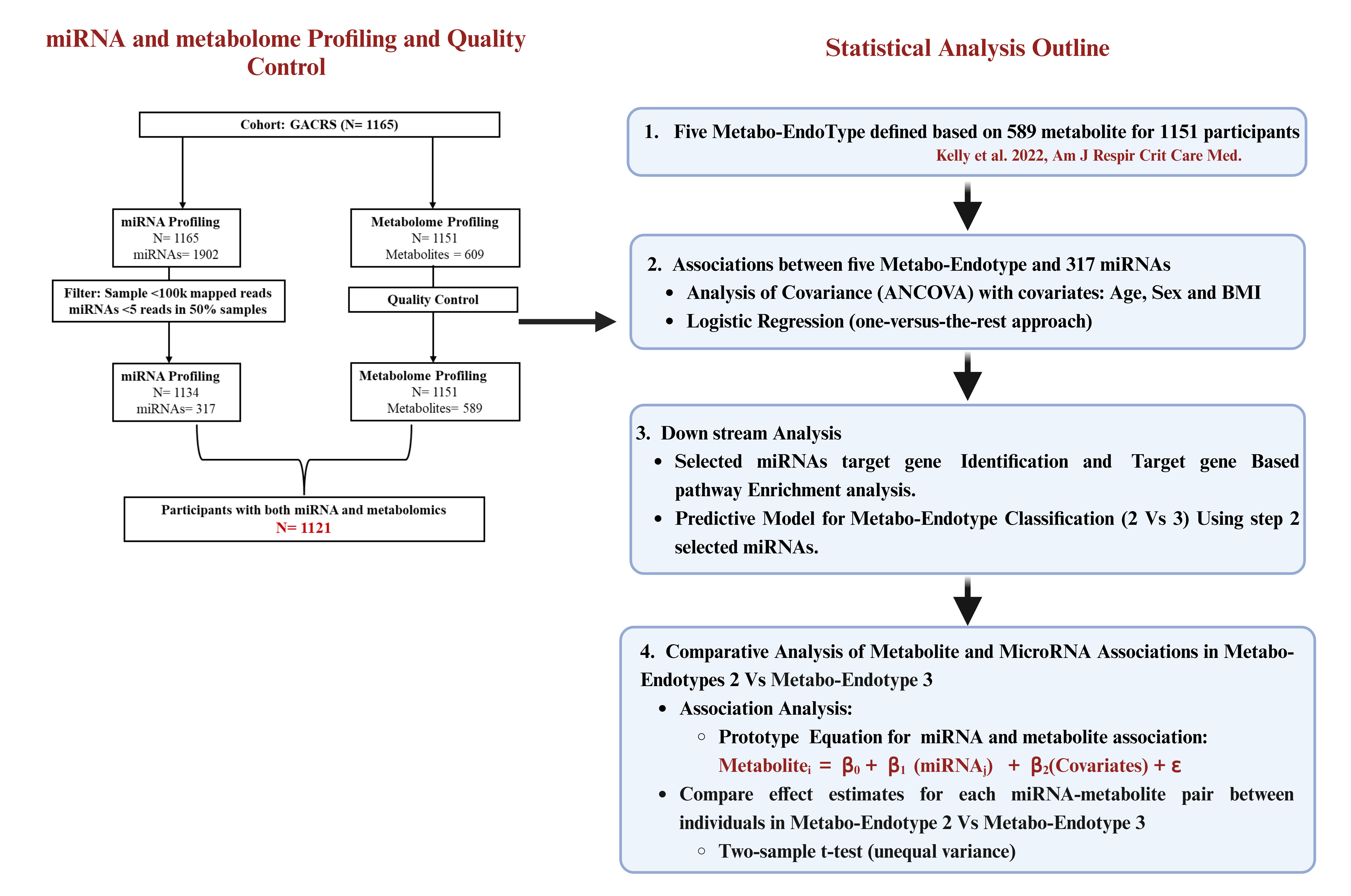


*Metabo-endotypes validated in* (6)

**Figure E2: GACRS batch effect check:** Guided principal component analysis (gPCA) was performed on mapped read counts to assess potential batch effects. The analysis showed no significant batch effect in the normalized data (p = 0.41). The PCA plot displays samples colored by sequencing batch, showing no evident separation between batches.


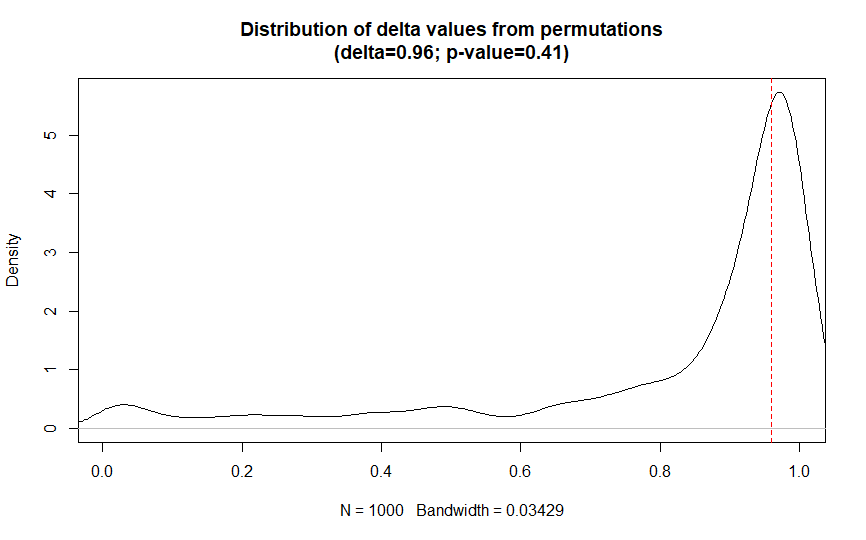

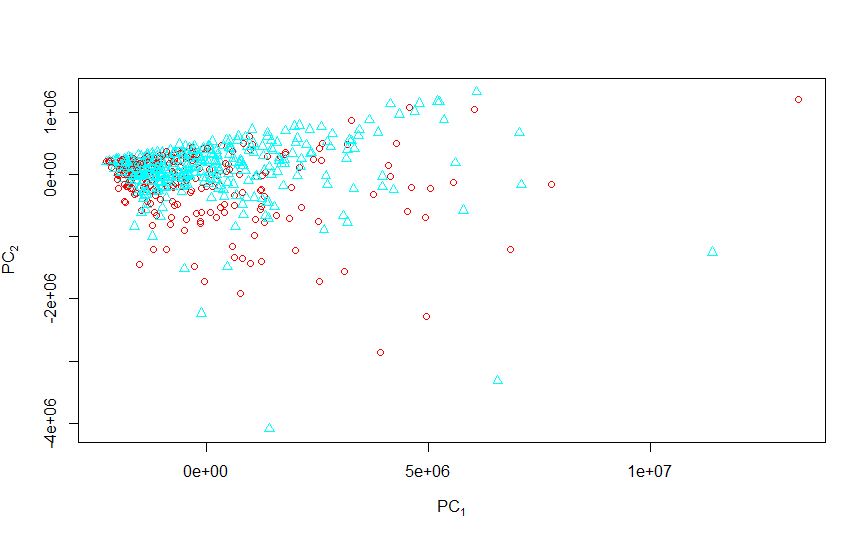


**Figure E3: Enrichment of** **123 miRNA with differing levels across five asthma metabo-endotypes and crossover in enriched genes**


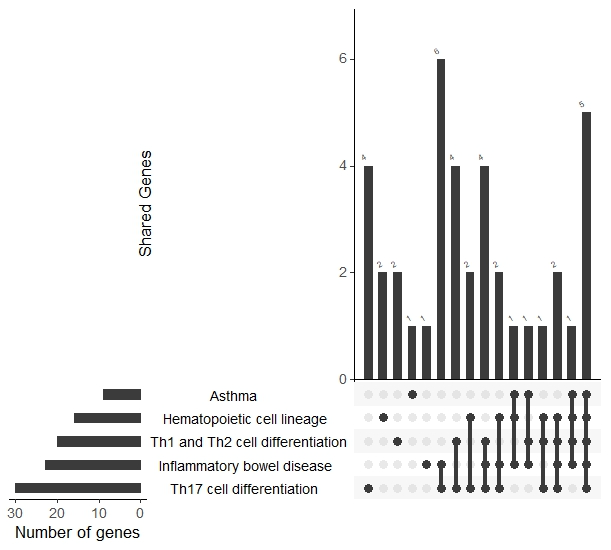


**Figure E4: Volcano plot of differential miRNA associations in the GACRS cohort.** Volcano plot of miRNAs from ANCOVA analysis in the GACRS cohort. The x-axis shows mean square expression values, and the y-axis shows –log₁₀ adjusted p-values. Significant miRNAs (adjusted p < 0.05) are highlighted in red; non-significant miRNAs are grey, emphasizing those with the largest and most significant group differences.


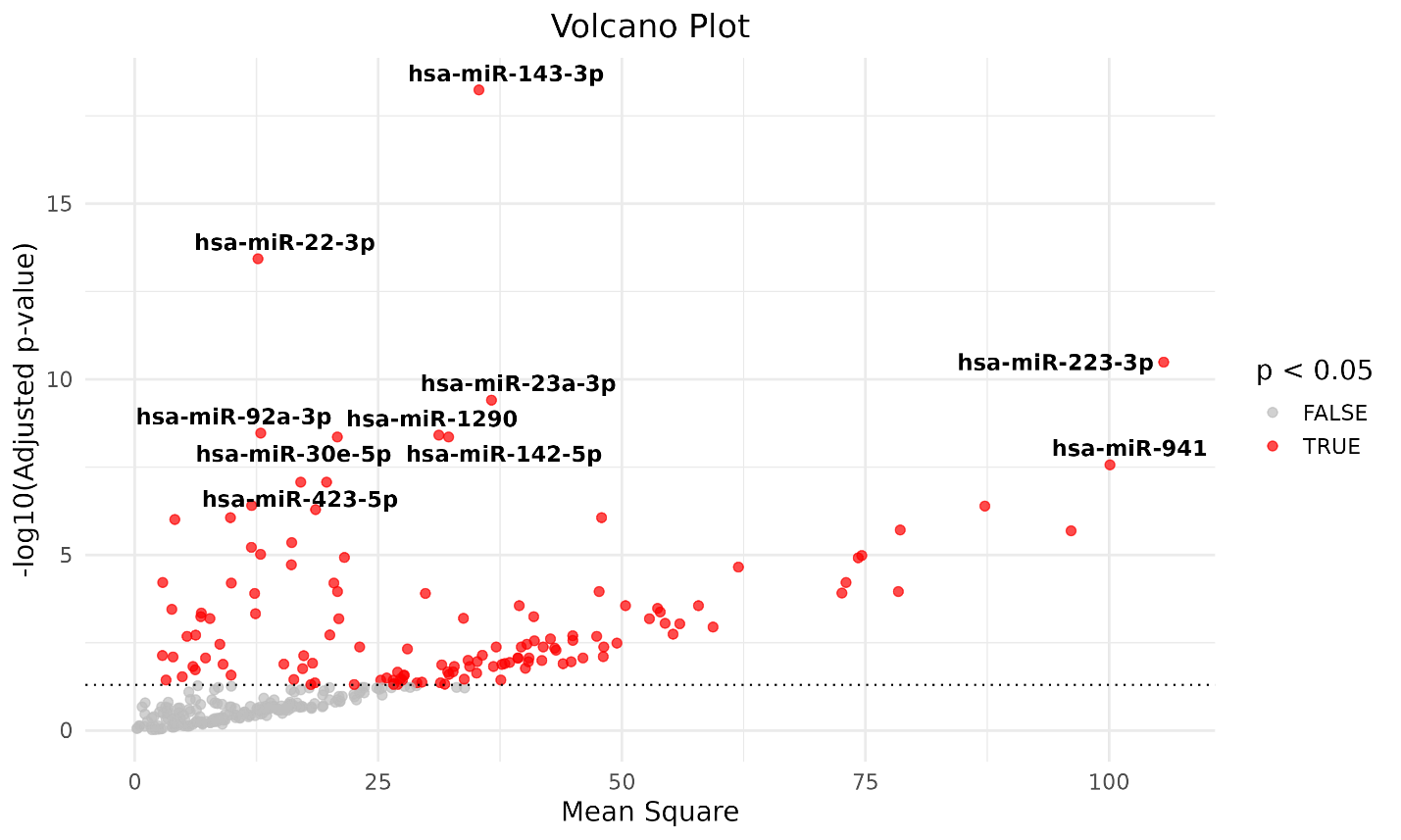


**Figure E5: Correlation analysis of the expression levels of 141 miRNAs identifies five clusters of highly correlated miRNAs**


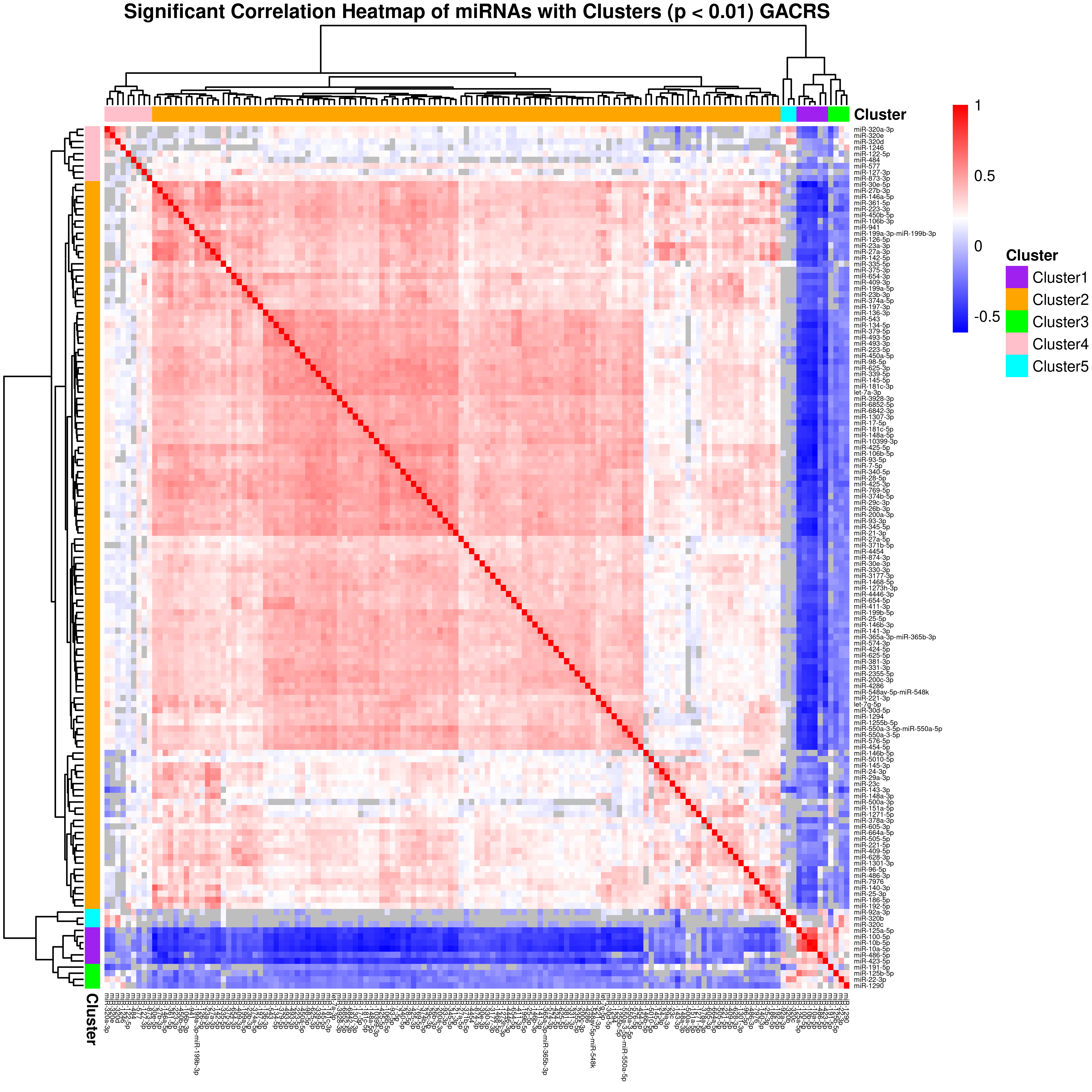


**Figure E6:** **Comparison of beta-coefficients from Metabo-endotypes 2 and 3 from the one-versus-the-rest regression models**
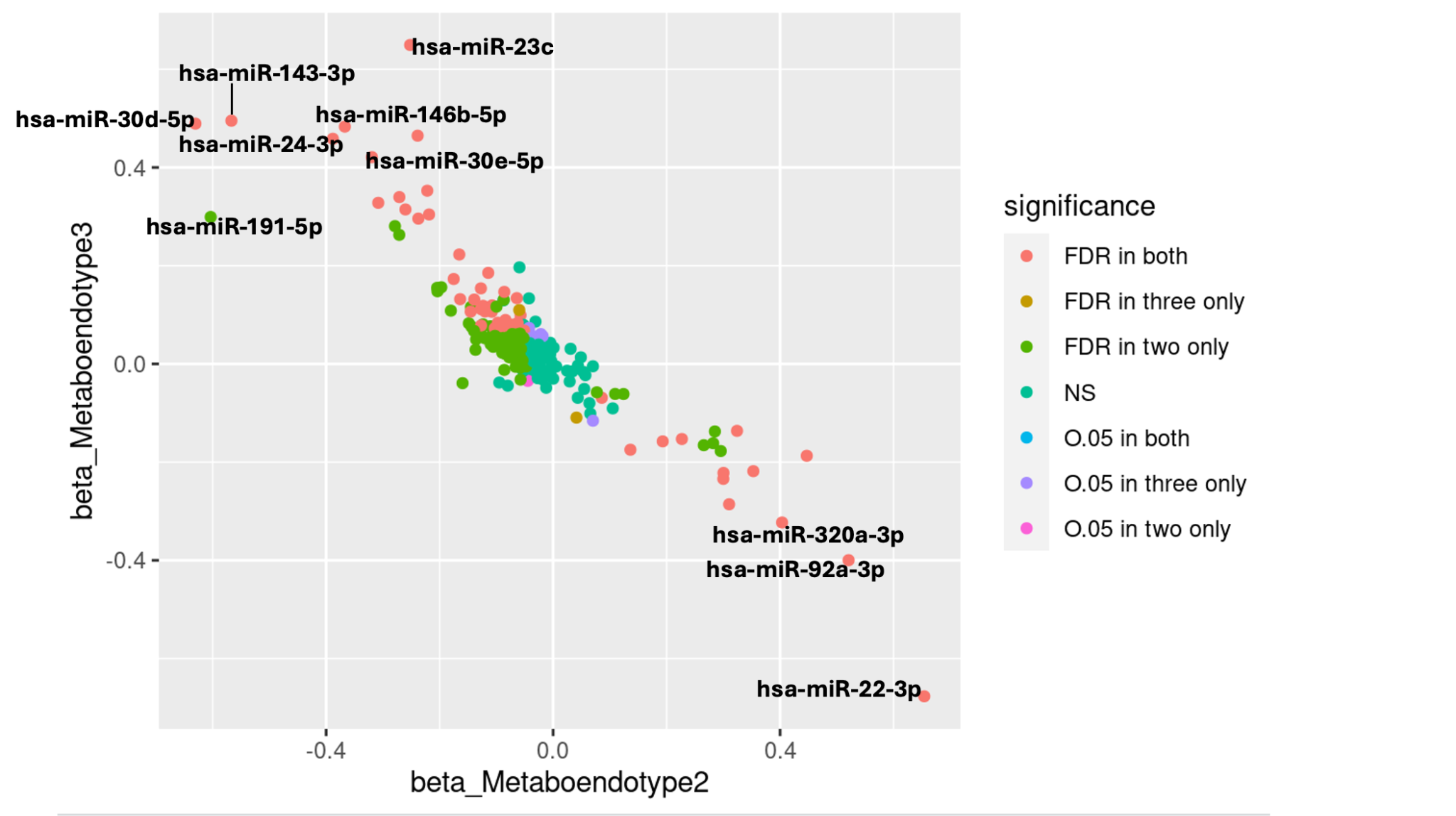


**Figure E7: Protein–protein interaction (PPI) network and KEGG pathway enrichment of target genes regulated by 17 LASSO-selected miRNAs**. (A) STRINGdb-derived PPI network of 336 target genes. (B) KEGG enrichment shows significant pathways related to inflammatory bowel disease, Th17 and Th1/Th2 cell differentiation, and other immune-inflammatory processes (FDR < 0.05). Circle size represents gene count; color intensity denotes significance.


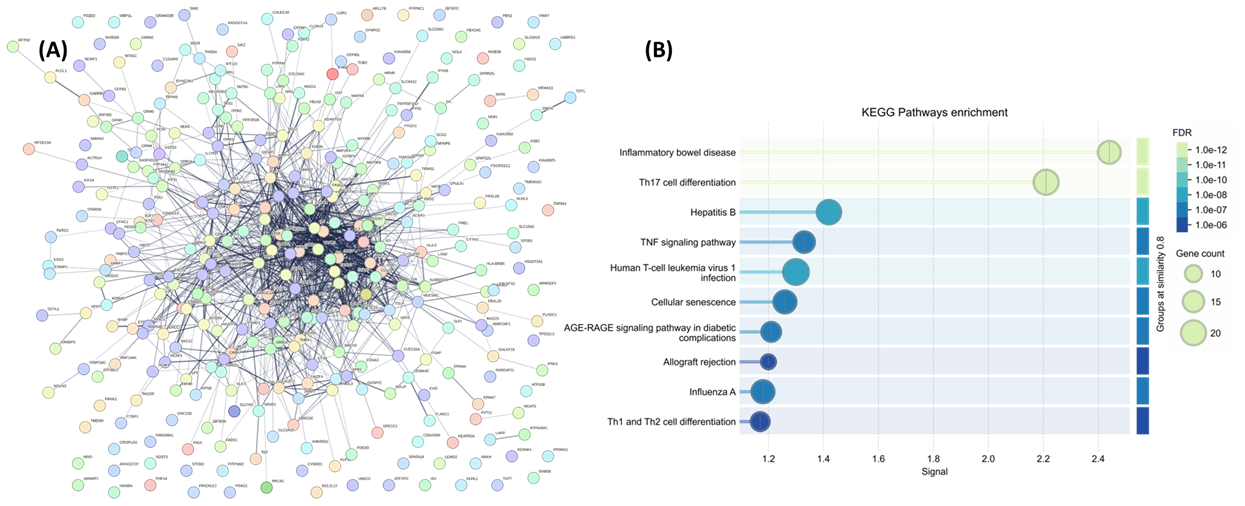


**Figure E8: Distribution of 1-methylguanine, arginine, hippurate and DG (38:4).**


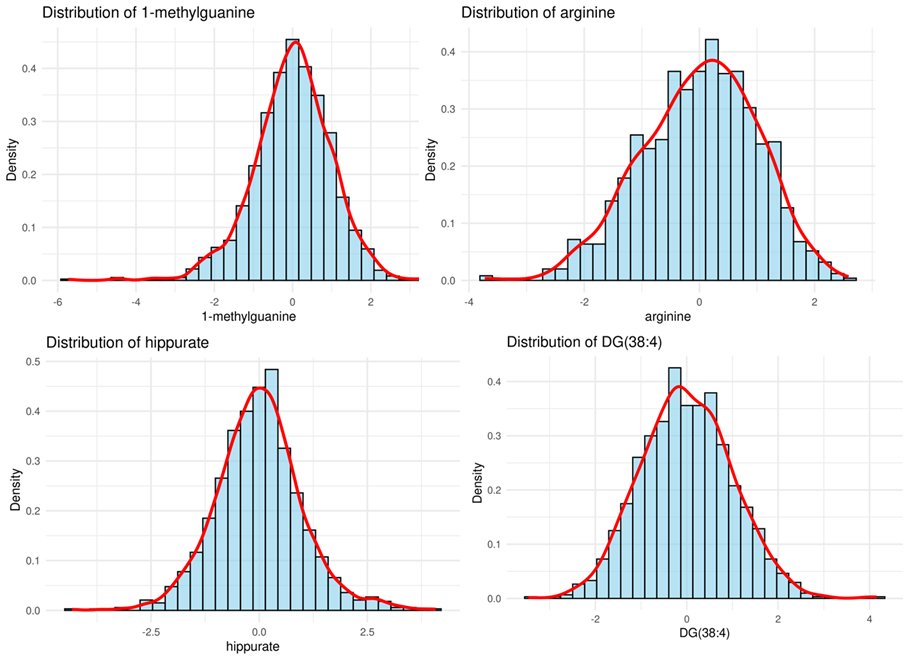


**Figure E9: Distribution and normality assessment of hsa-miR-143-3p expression.** Histogram with density overlay (left) and Q–Q plot (right) illustrating the distribution and normality of hsa-miR-143-3p expression levels. The histogram depicts the empirical density of expression values, while the Q–Q plot compares sample quantiles with theoretical normal quantiles.


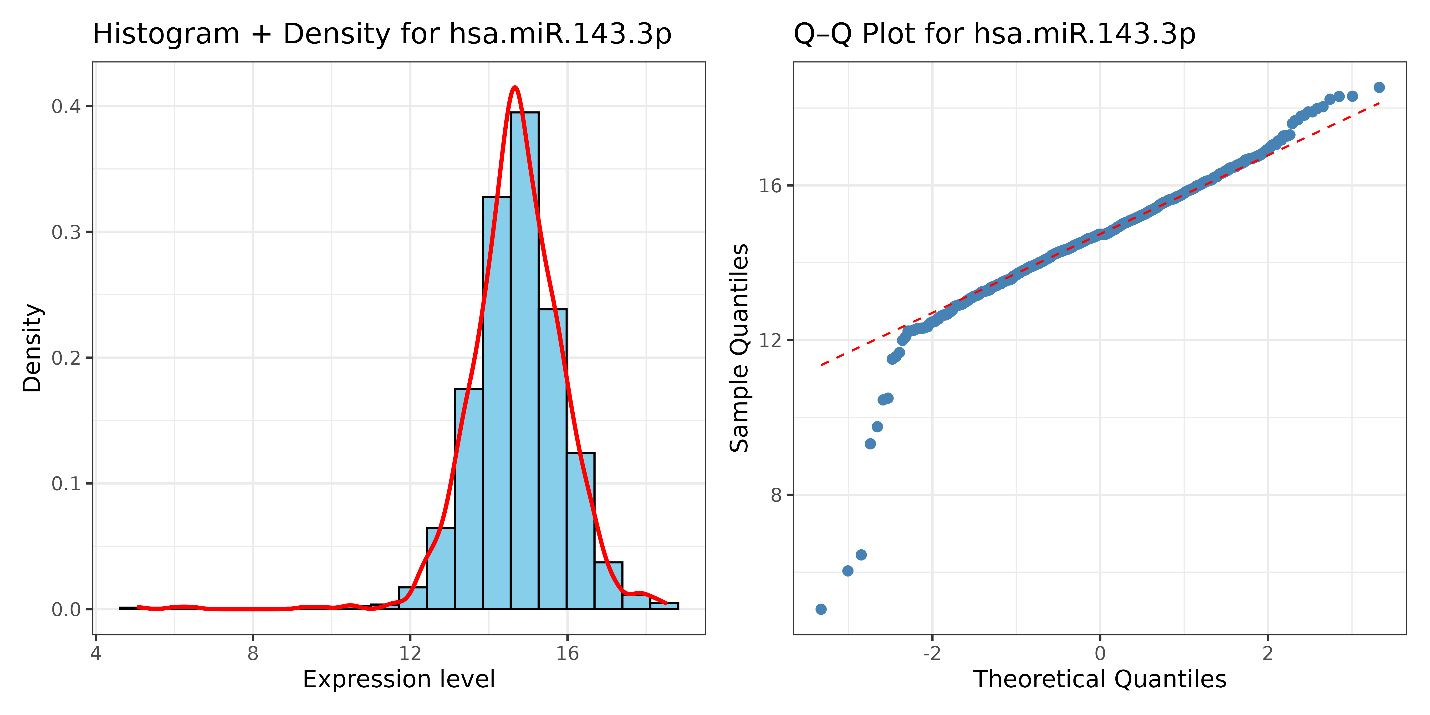


1. Kelly, R.S., Mendez, K.M., Huang, M., Hobbs, B.D., Clish, C.B., Gerszten, R., Cho, M.H., Wheelock, C.E., McGeachie, M.J., Chu, S.H., et al. (2022). Metabo-Endotypes of Asthma Reveal Differences in Lung Function: Discovery and Validation in Two TOPMed Cohorts. Am J Respir Crit Care Med *205*, 288-299. 10.1164/rccm.202105-1268OC.

2. Kelly, R.S., Virkud, Y., Giorgio, R., Celedon, J.C., Weiss, S.T., and Lasky-Su, J. (2017). Metabolomic profiling of lung function in Costa-Rican children with asthma. Biochim Biophys Acta Mol Basis Dis *1863*, 1590-1595. 10.1016/j.bbadis.2017.02.006.

3. A genome-wide search for asthma susceptibility loci in ethnically diverse populations. The Collaborative Study on the Genetics of Asthma (CSGA). (1997). Nat Genet *15*, 389-392. 10.1038/ng0497-389.

4. Hitchcock, D.S., Krejci, J.N., Sturgeon, C.E., Dennis, C.A., Jeanfavre, S.T., Avila-Pacheco, J.R., and Clish, C.B. (2024). <em>Eclipse</em>: A Python package for alignment of two or more nontargeted LC-MS metabolomics datasets. bioRxiv, 2023.2006.2009.544417. 10.1101/2023.06.09.544417.

5. Sumner, L.W., Amberg, A., Barrett, D., Beale, M.H., Beger, R., Daykin, C.A., Fan, T.W., Fiehn, O., Goodacre, R., Griffin, J.L., et al. (2007). Proposed minimum reporting standards for chemical analysis Chemical Analysis Working Group (CAWG) Metabolomics Standards Initiative (MSI). Metabolomics *3*, 211-221. 10.1007/s11306-007-0082-2.

6. Kowarik, A.T.M. (2016). Imputation with the R Package VIM. Journal of Statistical Software *74*.

7. Do, K.T., Wahl, S., Raffler, J., Molnos, S., Laimighofer, M., Adamski, J., Suhre, K., Strauch, K., Peters, A., Gieger, C., et al. (2018). Characterization of missing values in untargeted MS-based metabolomics data and evaluation of missing data handling strategies. Metabolomics *14*, 128. 10.1007/s11306-018-1420-2.
